# Triage of high-risk human papillomavirus-positive women by methylated *POU4F3*

**DOI:** 10.1186/s13148-015-0122-0

**Published:** 2015-08-21

**Authors:** Par Bahadur Pun, Yu-Ping Liao, Po-Hsuan Su, Hui-Chen Wang, Yu-Chih Chen, Yaw-Wen Hsu, Rui-Lan Huang, Cheng-Chang Chang, Hung-Cheng Lai

**Affiliations:** Molecular Cell Biology, Taiwan International Graduate Program, Academia Sinica, 128 Academia Road, Section 2, Nankang, Taipei, 11529 Taiwan; Graduate Institute of Life Science, National Defense Medical Center, No.161, Section 6, Min-Chuan East Road, Taipei, 11490 Taiwan; Department of Obstetrics and Gynecology, School of Medicine, College of Medicine, Taipei Medical University, No. 250, Wuxing Street, Taipei, 11031 Taiwan; Department of Obstetrics and Gynecology, Shuang Ho Hospital, Taipei Medical University, No. 291, Zhongzheng Rd., Zhonghe District, New Taipei City, 23561 Taiwan; Division of Research and Analysis, Food and Drug Administration, Ministry of Health and Welfare, No. 161-2, Kunyang St, Nangang District, Taipei, 11561 Taiwan; Department of Obstetrics and Gynecology, Tri-Service General Hospital, National Defense Medical, No. 161, Section 6, Min-Chuan East Road, Taipei, 11490 Taiwan

**Keywords:** DNA methylation, HrHPV test, QMSP, Biomarker, Cervical intraepithelial neoplasia (CIN), Cervical cancer screening

## Abstract

**Background:**

Insufficient specificity of the high-risk human papillomavirus (hrHPV) assay in primary cervical cancer screening results in unnecessary referral. Additional assays to triage hrHPV-positive women are needed to improve molecular cervical cancer screening. DNA methylation is a promising biomarker in cervical cancer. We evaluated the clinical performance of potentially methylated genes as a triage assay for hrHPV-positive women.

**Results:**

We conducted a retrospective hospital-based case–control study in Taiwan. Cervical scrapings were collected before colposcopy for hrHPV testing and quantitative methylation-specific PCR (QMSP) of 16 genes. Five genes, *POU4F3*, *HS3ST2*, *AJAP1*, *PAX1*, and *SOX1*, were prioritized for the clinical performance to triage hrHPV-positive women. Two hundred cervical scrapings were randomly classified into a training set (*n* = 111) and testing set (*n* = 89). All samples were tested for hrHPV using a Hybrid Capture II (HCII) assay. HrHPV-positive women were subjected to DNA methylation analysis by QMSP. In the training set, the receiver operating characteristic (ROC) curves defined the optimal methylation index (M-index) cutoff values for discriminating CIN3^+^ from CIN1/normal, which then were applied to the testing set. Among the five genes, *POU4F3* revealed the highest area under the ROC curve (AUC) (0.86; 95 % CI, 0.78–0.95) in detecting CIN3^+^. In the testing set, *POU4F3* revealed the best clinical performance in triage of hrHPV-positive women with a sensitivity of 74 % and specificity of 89 % for detecting CIN3^+^.

**Conclusions:**

*POU4F3* methylation analysis is a potential molecular tool for triage in detecting CIN3^+^ in hrHPV-positive women. The combined use of broad-spectrum HPV assay and *POU4F3* methylation analysis as a new generation of molecular cervical cancer screening warrants further population-based study.

**Electronic supplementary material:**

The online version of this article (doi:10.1186/s13148-015-0122-0) contains supplementary material, which is available to authorized users.

## Background

Cervical cancer is a common medical problem in women, with 528,000 new cases and 266,000 deaths globally in 2012 indicating the need to develop and implement an effective cancer screening strategy [1]. The Papanicolaou (Pap) smear for cytological examination has been used for the detection of precancerous cellular abnormalities of cervical cells for decades and has lessened the disease burden by reducing the mortality and morbidity of cervical cancer [2]. The Pap smear or cytology test has high specificity for cervical intraepithelial neoplasia; however, it has many drawbacks such as suboptimal sensitivity [3] and moderate accuracy to detect relevant lesions and subjective diagnosis of cervical abnormalities with poor reproducibility [4]. Oncogenic high-risk human papillomavirus (hrHPV) infection is a well-known etiology of cervical cancer [5]. Because the duration of the initial hrHPV infection until the development of invasive cancer is long, assay of HPV DNA as a screening tool is appealing [6, 7]. However, HPV infection is transient in nature, and only few infected lesions further progress as invasive cancer [8]. Insufficient specificity of the HPV DNA assay results in a high false-positive rate and extra medical burden because of the consequent high colposcopy referral rate [6]. Findings of HPV-positive assay results also cause adverse psychosocial impact [9]. Therefore, an additional triage assay is required to improve HPV-based molecular cervical cancer screening [10, 11].

Persistent oncogenic hrHPV infection causes genetic and epigenetic changes [12]. Promoter hypermethylation-mediated silencing of tumor suppressor genes is common in cervical carcinogenesis [12, 13]. Because DNA methylation can be easily quantitated using molecular methods, it is gaining attraction as a molecular assay for detecting cervical cancer [14]. Several studies including our group have revealed that numerous aberrantly DNA-methylated cervical cancer-related genes could be potential biomarkers to improve cervical cancer detection [12, 15–17], to triage women with atypical squamous cells [18, 19] and low-grade squamous intraepithelial lesions (LSILs) in Pap smears [20]. Methylated genes could be potential markers for the triage of hrHPV-positive women [21–27]. However, the sensitivity and specificity are not satisfactory even if combining two or more genes [23, 24, 26], highlighting the need for novel methylation biomarkers.

Using methylomic approaches, many methylated candidate genes have been revealed, including *ADRA1D*, *AJAP1*, *COL6A2*, *EDN3*, *EPO*, *HS3ST2*, *MAGI2*, *POU4F3*, *PTGDR*, *SOX8*, *SOX17*, *ST6GAL2*, *SYT9*, *ZNF614* [28], *SOX1*, and *PAX1* [29]. The performance of these methylated genes to triage hrHPV-positive women remains unexplored.

## Results

### Selection of potential candidate genes in hrHPV-positive women

We randomly collected cervical scrapings from 100 women including 20 normal, 20 CIN1, 20 CIN2, 20 CIN3/CIS, and 20 SCC/AC before treatment. Those samples from hrHPV-positive women, 67 out of 100, were subjected to quantitative methylation-specific PCR (QMSP) analysis of 14 genes, *ADRA1D*, *AJAP1*, *COL6A2*, *EDN3*, *EPO*, *HS3ST2*, *MAGI2*, *POU4F3*, *PTGDR*, *SOX8*, *SOX17*, *ST6GAL2*, *SYT9*, and *ZNF614*, and used the same cutoff values previously described [28] (Table 1). We selected candidate genes with a sensitivity of >85 % or specificity of >98 % in detecting CIN3^+^ in hrHPV-positive women for further validation. Three genes, *POU4F3*, *HS3ST2*, and *AJAP1*, fulfilled these criteria.

**Table 1:** Sensitivities and specificities of candidate genes in hrHPV-positive women (N = 67) in the selection set

Detection modality	Sensitivity (%)	Specificity (%)
ADRA1D	61	97
AJAP1	64	100
COL6A2	42	91
EDN3	58	97
EPO	67	97
HS3ST2	88	82
MAGI2	70	94
POU4F3	88	82
PTGDR	67	97
SOX8	46	91
SOX17	64	94
ST6GAL2	64	97
SYT9	73	94
ZNF614	58	97

### Generation of methylation cutoff values for triage of hrHPV-positive women in the training set

**Fig. 1:**
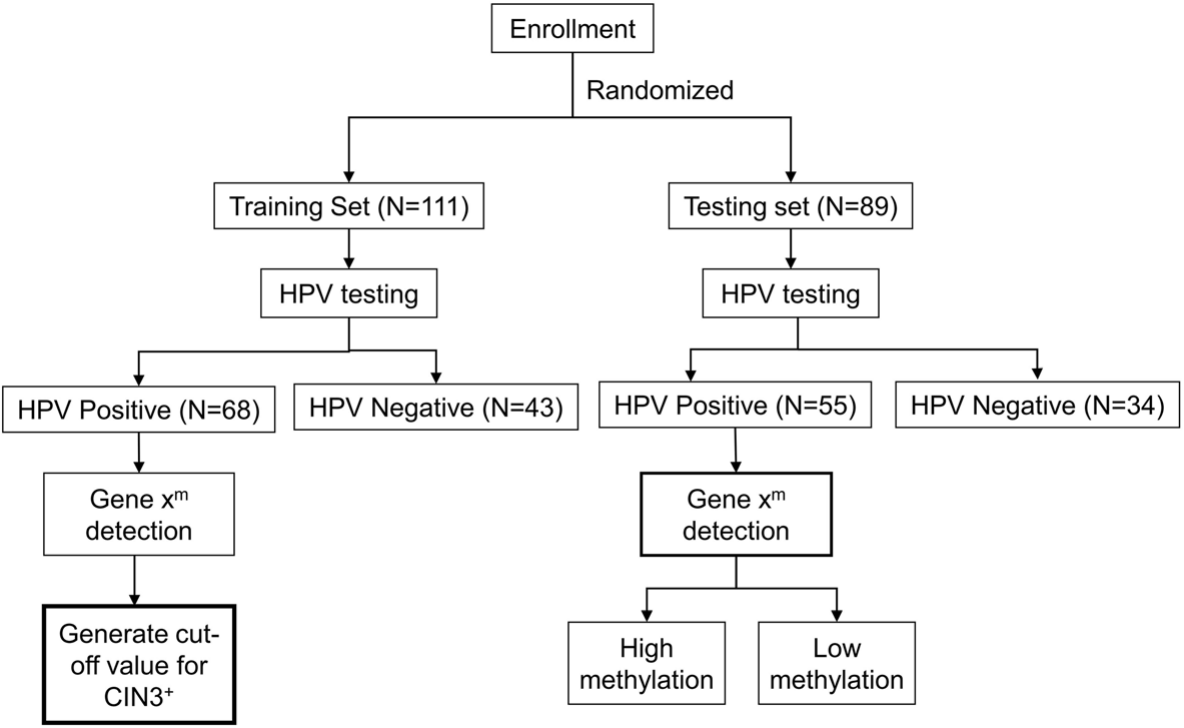
Work flow for analysis of clinical performance of candidate genes. A total of 200 women were enrolled and randomly assigned to a training set and a testing set. Methylation analysis of candidate genes using cervical scrapings of hrHPV-positive women under the training set was used for generating M-index cutoff values, which were then applied for analysis of the clinical performance of the candidate genes. X^m^ is the level of methylation of the candidate gene

**Fig. 2:**
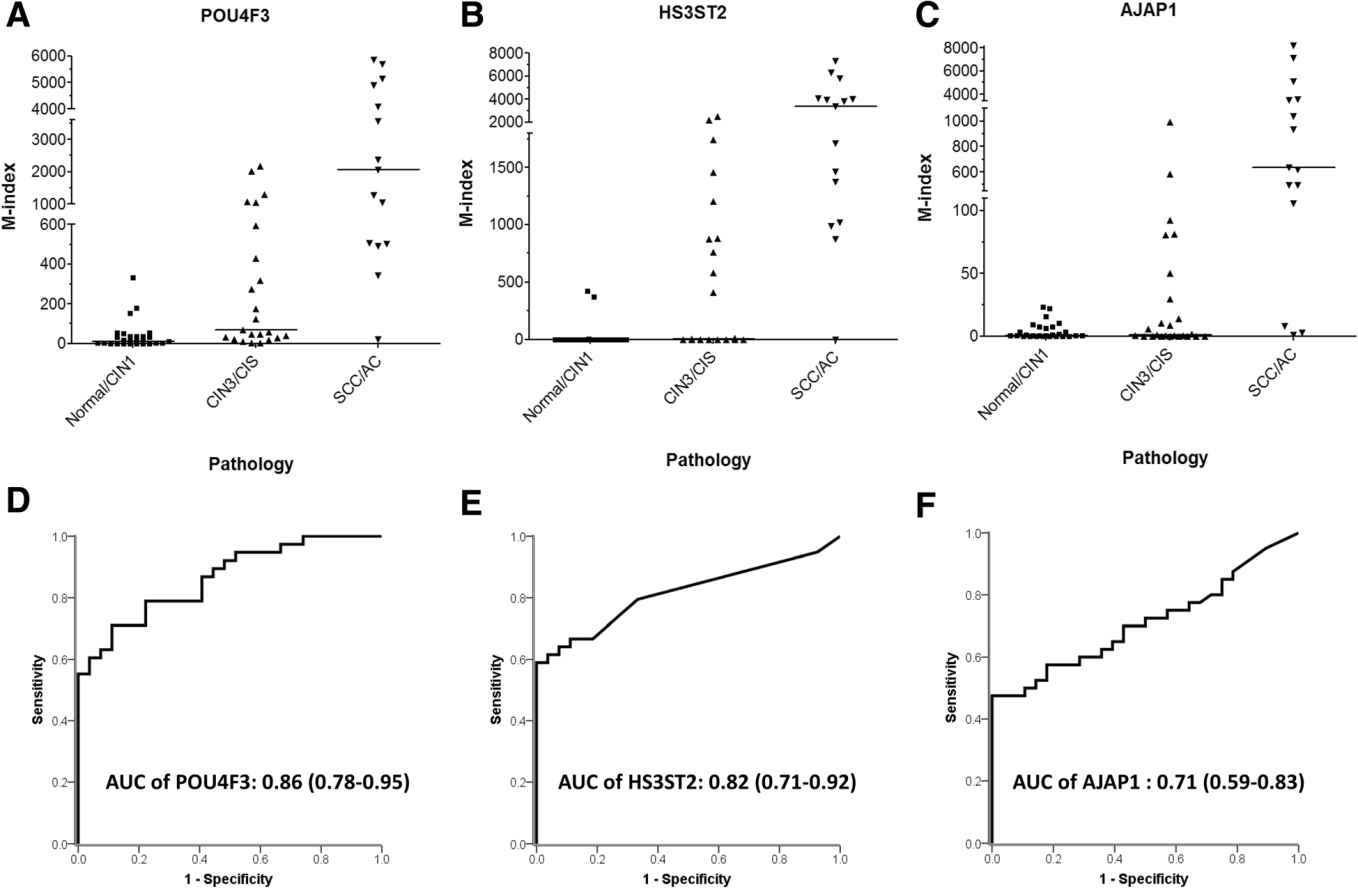
ROC curves of genes for M-index to trade off performance in detecting CIN3^+^. Methylation index levels of *POU4F3* (**a**), *HS3ST2* (**b**), and *AJAP1* (**c**) in cervical scrapings such as normal and tumors graded as normal/CIN1, CIN3/CIS, or SCC/AC diagnosed by proven histopathology in hrHPV-positive samples. Each *dot* in the figure represents the M-index level of an individual woman. Analysis of ROC curve of *POU4F3* (**d**), *HS3ST2* (**e**), and *AJAP1* (**f**). The AUC of the ROC curve of an individual candidate gene was calculated to diagnose CIN3^+^ lesions

We tested the clinical performance of *POU4F3*, *HS3ST2*, and *AJAP1* methylation for the triage of hrHPV-positive women (Fig. 1). The independently enrolled 200 women were randomly classified into two groups with a training-to-testing ratio of 1:1 (Table 2). There was no significant difference in the age (*P* = 0.17) and diagnosis distribution in the training set and testing set. Methylation levels of *POU4F3*, *HS3ST2*, and *AJAP1* in hrHPV-positive women increased with disease severity (Fig. 2a–c). The optimal methylation index (M-index) cutoff values for detecting CIN3^+^ were 38 for *POU4F3* and 2 for *HS3ST2* and *AJAP1* as defined by receiver operating characteristic (ROC) curves. The areas under the ROC curves (AUC) were 0.86 (95 % CI, 0.78–0.95) for *POU4F3*, 0.82 (95 % CI, 0.71–0.92) for *HS3ST2*, and 0.71 (95 % CI, 0.59–0.83) for *AJAP1* (Fig. 2d–f). Because we have previously discovered and tested *SOX1* and *PAX1* genes as potential biomarkers [29], we also included the data of these two genes in this study to compare their clinical performance. At the optimal M-index cutoff values, the sensitivities of *POU4F3*, *HS3ST2*, *AJAP1*, *SOX1*, and *PAX1* in discriminating CIN3^+^ among hrHPV-positive women were 79, 67, 63, 78, and 70 %, respectively, whereas the specificities were 78, 89, 64, 71, and 89 %, respectively (Table 3).

**Table 2:** Histopathology, mean age, and HPV percentage of the patients

Variable		Training set	Testing set
Age	Mean ± SD	46.8 ± 13.5	44.2 ± 13.3
		No (%)	No (%)
Result of pathology	Normal	34 (30.6)	31 (34.8)
	CIN1	31 (27.9)	19 (21.3)
	CIN2	0 (0)	0 (0)
	CIN3/CIS	28 (25.2)	27 (30.3)
	SCC/AC	18 (16.2)	12 (13.5)
HPV	Negative	43 (38.7)	34 (38.2)
	Positive	68 (61.3)	55 (61.8)
Total		111	89

*CIN* cervical intraepithelial neoplasia, *CIN1* CIN grade 1, *CIN2* CIN grade 2, *CIN3* CIN3 grade 3, *CIS* carcinoma in situ, *SCC* squamous cell carcinoma, *AC* adenocarcinoma, *HPV* human papillomavirus

**Table 3:** Performance of methylation biomarkers to detect CIN3^+^ in hrHPV-positive women at training and testing sets

		Gene name				
		POU4F3	HS3ST2	AJAP1	SOX1	PAX1
	M-index cutoff value	38	2	2	4	4
Training set (*N* = 68)	Sensitivity (%)	79	67	63	78	70
	Specificity (%)	78	89	64	71	89
	PPV (%)	83	90	71	80	90
	NPV (%)	72	65	55	69	68
Testing set (*N* = 55)	Sensitivity (%)	74	55	80	63	60
	Specificity (%)	89	100	74	74	100
	PPV (%)	93	100	85	82	100
	NPV (%)	64	56	67	52	58

*PPV* positive predictive value, *NPV* negative predictive value, *CIN3* ^*+*^ including CIN3/CIS, SCC/AC

### Validation of the clinical performance of methylated genes in the testing set

Cervical scrapings of 55 hrHPV-positive women out of 89 women were analyzed further in the testing set for DNA methylation levels (Fig. 1). The testing set validated that *POU4F3* methylation analysis conferred the best clinical performance among five potential candidates with 74 % sensitivity and 89 % specificity (Table 3). When stratified by histology, *POU4F3* and *AJAP1* methylation testing did not miss any invasive cancer patients (Table 4). *AJAP1* methylation had better performance in detecting CIN3/CIS lesions than *POU4F3* (70.8 vs. 62.5 %). However, more CIN1 lesions were detected using *AJAP1*.

**Table 4:** Clinical performance of methylation biomarker in hrHPV-positive women of testing set stratified by histology

Total number of detectable		53	52	54
Detection modality		Gene name		
		*POU4F3*	*HS3ST2*	*AJAP1*
		Methylation positive/total number (%)		
Result of pathology	Normal	0/5 (0 %)	0/5 (0 %)	0/5 (0 %)
	CIN1	2/13 (15.4 %)	0/14 (0 %)	5/14 (35.7 %)
	CIN3/CIS	15/24 (62.5 %)	8/22 (36.3 %)	17/24 (70.8 %)
	SCC/AC	11/11 (100 %)	10/11 (90.9 %)	11/11 (100 %)
	Total	28	18	33

*CIN* cervical intraepithelial neoplasia, *CIN1* CIN grade 1, *CIN3* CIN3 grade 3, *CIS* carcinoma in situ, *SCC* squamous cell carcinoma, *AC* adenocarcinoma

## Discussion

Previous studies support the concept that DNA methylation could be a potential molecular biomarker for detection of cervical lesions [12, 15, 16, 28, 30]. An ideal methylation biomarker should have better specificity than HPV testing and better sensitivity than cytology when applied as a primary screening tool. Recent studies proposed the alternative role of methylation biomarkers as a triage method for hrHPV-positive women [22–24, 26, 27]. More high-risk HPV genotype detection may have a better chance to include more women at risk for triage in the primary screening. In addition, the distribution of HPV type varies across continents because 16, 31, 33, and 18 are prevalent in Europe, and 16, 58, 52 and 18 are prevalent in the Asia–Pacific region [31, 32]. We used the Hybrid Capture II (HCII) assay for hrHPV testing, which assays 13 high-risk genotypes simultaneously [7, 32, 33]. The present study demonstrated that DNA methylation analysis as a triage for hrHPV-positive women is feasible. The *POU4F3* methylation analysis confers the best clinical performance when combined with the HCII assay. In this study, the primary objective was to use broad-spectrum hrHPV testing capable of detecting more susceptible women for further triage with *POU4F3* methylation to achieve a better sensitivity. Further hrHPV subtype analysis may clarify type-specific correlation with *POU4F3* methylation, which may be useful in estimating the impact of molecular screening strategy using HPV detection followed by methylation triage in post-vaccination era.

*POU4F3* is located on chromosome 5q32 and plays various biological functions, such as regulation of transcription, cellular and metabolic processes, organ development, cellular differentiation, nervous system development, neurogenesis, and generation of neurons [34]. The function of *POU4F3* in cancer biology remains largely unknown. *POU4F3* hypermethylation in cervical cancer and glioma suggests its suppressor role in cancer [28, 34]. This study supports the concept that *POU4F3* could be a potential triage biomarker for hrHPV-positive women.

**Fig. 3:**
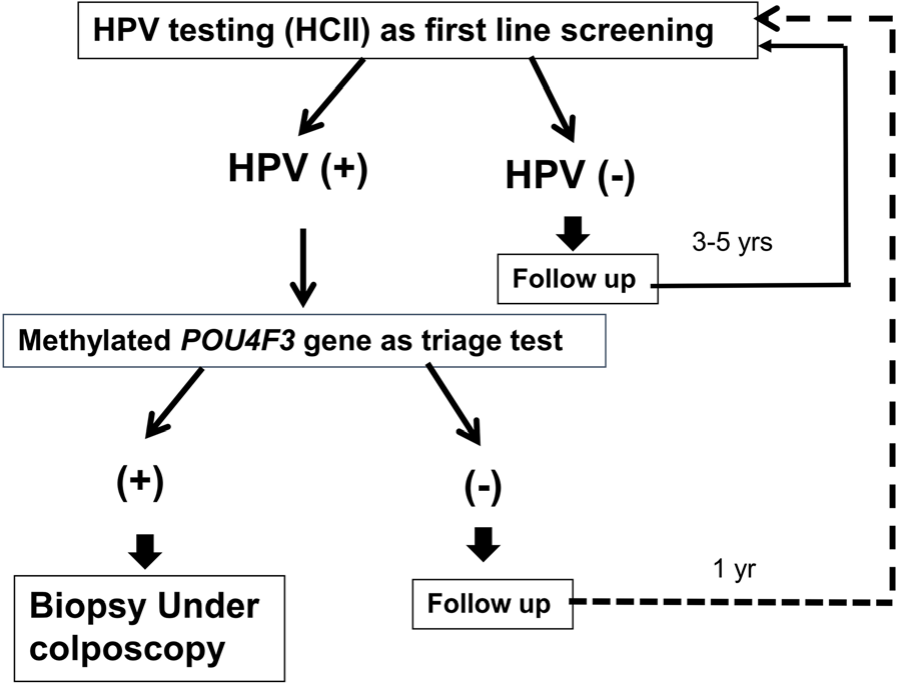
Proposed cervical cancer screening strategy using hrHPV assay and *POU4F3* methylation analysis as a triage test. In this proposed scenario, HCII hrHPV DNA assay is used as the primary screening test, where women without hrHPV infection undergo follow-up 3 to 5 years later. Samples from women with hrHPV infection undergo *POU4F3* methylation analysis, where women having positive *POU4F3* methylation are referred for colposcopy. Additionally, women with a positive hrHPV assay but negative *POU4F3* methylation may repeat HPV assay and DNA methylation analysis 1 year later

In the present study, a single gene, *POU4F3*, has a specificity of 89 % in detecting CIN3^+^ in hrHPV-positive women with limited compromise in sensitivity (79 to 74 %), which is better than the specificities previously published using *FAM19A4* (67 %) [27], or a panel of two genes *CADM1–M18/MAL-M1* (71–83 %) [22, 23, 35, 36], or a panel of at least two out of five methylated biomarkers (77 %) [26], or a panel with four methylated biomarkers (69 %) [24] or comparable to the specificity of *JAM* (88 %) [37]. We propose a scenario for the combination of HPV assay and *POU4F3* methylation analysis for cervical cancer screening (Fig. 3). However, it requires further independent validation together with additional standalone biomarker. Women without hrHPV infection undergo follow-up 3 to 5 years later [38]. Women with hrHPV infection will undertake *POU4F3* methylation analysis. Women having positive *POU4F3* methylation are referred for colposcopy. Because *POU4F3* methylation analysis did not miss any invasive cancer, *POU4F3* methylation-negative, hrHPV-positive women may repeat HPV assay and DNA methylation analysis 1 year later. This strategy may substantially reduce the referral rate. However, a longitudinal follow-up study is needed to clarify the natural history of those infected with hrHPV, but without *POU4F3* hypermethylation to determine if a longer interval between screenings is also safe. The high negative predictive value in hrHPV-negative women is well documented, which means a longer screening interval is safe. However, determining POU4F3 methylation in HPV-negative women to assess *POU4F3* as independent from HPV as a marker for cervical neoplasia/CIN/CIN3/cancer may also be a consideration. Because HPV assay and methylation analysis can be conducted in the same self-collected cervical sample, the application of this approach may improve the participation of women for screening [39, 40], especially those in low-resource areas. The application of a DNA methylation analysis using self-collected vaginal samples warrants further evaluation. In addition, this is a retrospective hospital-based study, which did not follow up the participants. Population-based studies in different geographical and ethnic backgrounds are needed to validate these results.

In the present study, we adapted histopathologically diagnosed CIN3^+^ as the end point because CIN2 is equivocal in nature with a tendency to regress to normal instead of progressing to CIN3^+^, where the likelihood of CIN2 progression to invasive cancer is only 5 % [41]. Further, diagnosis of CIN2 is much less reproducible than CIN3 because of the difference in the natural history of CIN2 from that of CIN3 [42]. However, CIN3 has a higher tendency to progress to invasive cancer because it is an immediate precursor with a similar virological profile and has better reproducibility [31]. Therefore, it is more appropriate to adapt CIN3^+^ as a surrogate end point for early diagnosis of cervical cancer.

## Conclusions

*POU4F3* methylation testing is a potential molecular biomarker for the triage of hrHPV-positive women for CIN3^+^ lesions. We envision an era of molecular screening for cervical cancer.

## Methods

### Patients

We conducted a retrospective case–control study using hospital-based patient samples in the Tri-Service General Hospital, Taiwan, from December 2009 to November 2010. Patients aged ≥20 years referred for a colposcopy and cervical biopsy and who were managed with conization or surgery after biopsy revealing CIN3^+^ were enrolled in this study. Cervical scrapings for laboratory analysis were collected in sterile phosphate-buffered saline before biopsy using a cervical brush and were stored at 4 °C until DNA extraction for HPV testing using a HCII hrHPV DNA assay (Digene, Silver Spring, MD, USA) and quantitative DNA methylation analysis of potential candidate genes using QMSP. Healthy women undergoing routine Pap screening were selected as controls, only when their Pap smears showed normal pattern. Women with positive or suspicious Pap smears were excluded from control. Before the study, all the subjects were informed about the study and were enrolled after obtaining documented full consent. Final diagnosis regarding different stages of cancer was performed by tissue-proven histopathological examination, except in healthy control women. Exclusion criteria applied in this study were compromised quality of Pap smears, patients previously vaccinated with anti-HPV vaccine, cervical neoplasia or existence of other malignancies, surgery related to the uterine cervix, an immunocompromised state, genital warts, or pregnancy. Further, all specimens were delinked from clinical information after numbering each of them until data analysis. In this study, all the women were tested for HPV infection and only samples from hrHPV-positive women underwent DNA methylation analysis. Cervical scrapings of 67 hrHPV-positive women among 100 recruited women underwent DNA methylation analysis to prioritize candidate genes for further analysis of clinical performance. Cervical scrapings from 200 women recruited for analysis of clinical performance were randomly classified using a random number table as a 1:1 ratio into a training set (*n* = 111) and a testing set (*n* = 89). The training set included 46 women with histopathologically confirmed CIN3^+^ and 65 women with normal/CIN1. Cervical scrapings from 68 hrHPV-positive women of the 111 underwent DNA methylation analysis. Methylation levels in the training set were used to generate optimal M-index cutoff values of candidate genes that can distinguish relevant cancerous cases from control. The clinical performance of candidate genes was validated using the optimal cutoff values in the testing set. The testing set comprised 89 women including 39 women with CIN3^+^ and 50 women with normal/CIN1. Of the 89 women, cervical scrapings from 55 hrHPV-positive women were analyzed further for quantitative assay of DNA methylation. This study was approved by the Institutional Review Board of the Tri-Service General Hospital, National Defense Medical Center.

### Extraction of DNA followed by bisulfite modification

Genomic DNA was extracted as previously described using a DNeasy Blood & Tissue Kit (Qiagen, Hilden, Germany) according to the manufacturer’s recommendations [28]. Those samples with a DNA yield as low as 500 ng or more (>500 ng) as measured by NanoDrop ND-1000 (Thermo Scientific, Wilmington, DE, USA) were considered for further analysis in this study. Bisulfite modification of genomic DNA samples was performed using a CpGenome DNA Modification Kit (Millipore, Temecula, CA, USA) according to the manufacturer’s recommendations, and the samples were dissolved in 70 μL of nuclease-free water [29]. Bisulfite-converted DNA was stored at −80 °C until further use.

### Methylation assays of potential candidate genes

QMSP used for analysis of the methylation status of the candidate genes was based on the principle of fluorescence-based real-time PCR. TaqMan-based QMSP amplification was performed on the bisulfite-treated DNA [43]. The type II collagen gene (*COL2A*) was used as an internal reference. In vitro methylated genomic DNA treated with CpG methyltransferase (M.SssI; New England Biolabs, Beverly, MA, USA) was used as a positive control. While prioritizing potential candidate methylated genes for further performance analysis, QMSP was performed in a TaqMan probe system using an Applied Biosystems 7900HT Fast Real-Time PCR System in a total volume of 20 μL reaction mixture containing 2 μL of bisulfite template DNA, 250 nM of each primer, 225 nM TaqMan probe, and 10 μL of FastStart Universal Probe Master (ROX) (Roche Diagnostics, Roche Applied Science, Mannheim, Germany) [28]. 6-Carboxy-fluorescein was used to label the 5′ end of probes, while a quencher dye (MGB by Applied Biosystems, or BHQ1 by TIB) was used to label the 3′ end of the probes (Additional file 1: Table S1). However, for analysis of clinical performance of candidate genes, QMSP for *AJAP1*, *HS3ST2*, and *POU4F3* and multiplex QMSP for *PAX1* and *SOX1* were performed in a TaqMan probe system using a LightCycler 480 Real-Time PCR System (Roche Diagnostics, Roche Applied Science) [29]. Briefly, the total reaction volume of 20 μL contained 2 μL of modified template DNA, 1 μL of 20× Custom TaqMan reagent, and 10 μL LightCycler 480 Probes Master (Roche Diagnostics, Roche Applied Science). A mixture of primers and probes was used for *PAX1* and *SOX1*. The reactions were conducted using an initial incubation at 95 °C for 10 min, followed by 50 cycles of 95 °C for 10 s, and annealing and extension for 40 s at 60 °C (using the thermal cycler protocol in the standard mode). The level of DNA methylation was measured in terms of M-index [30]. Results showing the very high Cp values of *COL2A* (>36) were defined as detection failures.

### HPV DNA assay

The HCII hrHPV DNA assay (Digene) was used as the primary assay in this study following the manufacturer’s protocol to detect hrHPV infection. This HCII assay can detect 13 high-risk HPV subtypes: 16, 18, 31, 33, 35, 39, 45, 51, 52, 56, 58, 59, and 68. Samples with a ratio of relative light units (RLU)/cutoff value higher than 1.0 were recorded as positive.

### Statistical analysis

ROC curves for each of the candidate genes were calculated using the data from the training set. Optimal M-index cutoff values of the candidate genes were generated from ROC curves and were used to further analyze the clinical performance of the candidate methylated genes in the testing set. Sensitivities, specificities, positive predictive values (PPV), and negative predictive values (NPV) of *AJAP1*, *HS3ST2*, *POU4F3*, *PAX1*, and *SOX1* for detecting CIN3^+^ were calculated. IBM SPSS Statistics for Windows, version 20.0 (Armonk, NY, USA) was used for all statistical analyses.

## Additional file

Additional file 1: Table S1. QMSP primers and probes in this study.

## Abbreviations

*ADRA1D*: adrenoceptor alpha 1D
*AJAP1*: adherens junctions-associated protein 1
AUC: area under the receiver operating characteristic curve
CIN: cervical intraepithelial neoplasia
CIN3^+^: CIN grade 3 or worse
*COL6A2*: collagen, type VI, alpha 2
*EDN3*: endothelin 3
*EPO*: erythropoietin
HrHPV: high-risk human papillomavirus
*HS3ST2*: heparan sulfate (glucosamine) 3-*O*-sulfotransferase 2
*MAGI2*: membrane-associated guanylate kinase, WW and PDZ domain-containing 2
*PAX1*: paired box gene 1
*POU4F3*: POU class 4 homeobox 3
*PTGDR*: prostaglandin DP receptor
QMSP: quantitative methylation-specific PCR
*SOX1*: sex-determining region Y box 1
*SOX17*: SRY-box 17
*SOX8*: SRY (sex-determining region Y)-box 8
*ST6GAL2*: ST6 betagalactosamide alpha-2,6-sialyltranferase 2
*SYT9*: synaptotagmin IX
*ZNF614*: zinc finger protein 614
