# Supplementary material for: Triage of high-risk human papillomavirus-positive women by methylated POU4F3
Source: Clin Epigenetics. 2015 Aug 21;7(1):85. doi: 10.1186/s13148-015-0122-0 (PMC4546171; doi:10.1186/s13148-015-0122-0)
Supplement: Additional file 1: Table S1. — QMSP primers and probes in this study. [file 13148_2015_122_MOESM1_ESM.docx]

**Table S1 QMSP primers and probes in this study**

| Gene | Forward Primer Sequence | Reverse Primer Sequence | Probe Oligo Sequence |
| --- | --- | --- | --- |
| *ADRA1D* | GGT TAG GTA GTT TCG TTT TCG GAT AGT C | AAA CAC AAA ACG AAC GAC CGA CA | 6FAM-TAACCGCGAAACTCCAA-MGB |
| *AJAP1* | TTT GGT AGA GTT TTT CGA TTC GGT AGC | ACC GAA ACT CCG CGC CGA TAA | 6FAM-AACGAACGCGAATCCCCTC-MGB |
| *COL6A2* | TTT TAG GGT TTT CGT CGG TTT TGC | CGC CAA CCC CTA CCC GCT AC | 6FAM-ACTCGAACTTCGCGTCCC-MGB |
| *EDN3* | GGT AGC GCG TTT TGA AAG TTT ATG ATC | TAA ATC AAA CGC CGA AAC ACC GA | 6FAM-CGACCGCTTATAACCGC-MGB |
| *EPO* | GTT TTT GGG TTA TTT CGG TCG TTC | AAA AAA CGA CTA TCC AAA AAA CGC | 6FAM-CGATAACCCCGATCCGACTCCGAA-BHQ1 |
| *HS3ST2* | GTA AGA GTT TGG GAG CGT TCG AGT C | CAA AAA ATC CCG AAA ACA ACG AC | 6FAM-CCAACATCTCCCGATCC-MGB |
| *MAGI2* | CGT AGA GTT CGA GAT GTG GTA TTA GGC | AAA CTC CTA TAC GAA AAA AAC GCG CTA | 6FAM-CCGATAAAACAAAAATAACG-MGB |
| *POU4F3* | AGC GCG GGC GTT GAG TAG C | CGC GCT CCT AAC AAA ATA ACA ACG AA | 6FAM-TAACTTACTTACCGCTCTCC-MGB |
| *PTGDR* | TTG TTT CGC GTT TTT TAA TGT TAG C | AAA AAA ACT CCG AAA ACG ACG AAA T | 6FAM-CACGACAAAAACCTCCTAT-MGB |
| *SOX8* | GGG TTT CGT TTT CGG GGG ATC | ATC CCG CTA CGA AAC CCG CAA CA | 6FAM-CCCCGCGCCCGACTA-MGB |
| *SOX17^1^* | GGA GAT TCG CGT AGT TTT CG | AAC CCG ACC ATC ACC GCG | 6FAM-CGCGCTCTAAATCTAACTC-MGB |
| *ST6GAL2* | GGT GGG GGT TTT TAG GGA GTA TCG TTA TC | CGA CTA AAT AAA ACG CAA AAA AAC GCA AC | 6FAM-CCGACTCGCTAACTCC-MGB |
| *SYT9* | TGG GGT CGT CGT TAT TTT ATT TTG C | CCG CCC GAT CCC TCC GTC | 6FAM-AACGATACCGCACAACCCA-MGB |
| *ZNF614* | TTT CGG GTA GGT TTC GTG GTT TTC | CTA ACA ACG ACA AAA CGC CGC ATC | 6FAM-CCGAAAACGCTTCCTATT-MGB |
| *COL2A1*^2^ | GGG AAG ATG GGA TAG AAG GGA ATA T | TCT AAC AAT TAT AAA CTC CAA CCA CCA A | 6FAM-TTCATTCTAACCCAATACCT-MGB |

^1^ *SOX17* primer was referenced from Fu DY et al. Breast Cancer Res Treat. 2010. 119(3):601-612.

^2^ *COL2A1* was used as an internal reference gene by amplifying non-CpG sequences.

Note: QMSP primers and probe sequences for *PAX1* and *SOX1* will be provided upon request.
